# Machine learning empowered COVID-19 patient monitoring using non-contact sensing: An extensive review

**DOI:** 10.1016/j.jpha.2021.12.006

**Published:** 2022-01-04

**Authors:** Umer Saeed, Syed Yaseen Shah, Jawad Ahmad, Muhammad Ali Imran, Qammer H. Abbasi, Syed Aziz Shah

**Affiliations:** a Research Centre for Intelligent Healthcare, Coventry University, Coventry, CV1 5FB, UK; b School of Computing, Engineering and Built Environment, Glasgow Caledonian University, Glasgow, G4 0BA, UK; c School of Computing, Edinburgh Napier University, Edinburgh, EH11 4BN, UK; d James Watt School of Engineering, University of Glasgow, Glasgow, G12 8QQ, UK

**Keywords:** Artificial intelligence, Non-invasive healthcare, Machine learning, Non-contact sensing, COVID-19

## Abstract

The severe acute respiratory syndrome coronavirus 2 (SARS-CoV-2), which caused the coronavirus disease 2019 (COVID-19) pandemic, has affected more than 400 million people worldwide. With the recent rise of new Delta and Omicron variants, the efficacy of the vaccines has become an important question. The goal of various studies has been to limit the spread of the virus by utilizing wireless sensing technologies to prevent human-to-human interactions, particularly for healthcare workers. In this paper, we discuss the current literature on invasive/contact and non-invasive/non-contact technologies (including Wi-Fi, radar, and software-defined radio) that have been effectively used to detect, diagnose, and monitor human activities and COVID-19 related symptoms, such as irregular respiration. In addition, we focused on cutting-edge machine learning algorithms (such as generative adversarial networks, random forest, multilayer perceptron, support vector machine, extremely randomized trees, and k-nearest neighbors) and their essential role in intelligent healthcare systems. Furthermore, this study highlights the limitations related to non-invasive techniques and prospective research directions.

## Highlights

• This article describes cutting-edge technology (invasive/non-invasive) and its role in the recognition of COVID-19 symptoms.
• This article summarizes state-of-art machine-learning algorithms and their roles in modern healthcare systems.
• This article presents the challenges associated with wireless sensing techniques and potential future research directions.

## Graphical abstract

**Figure:**
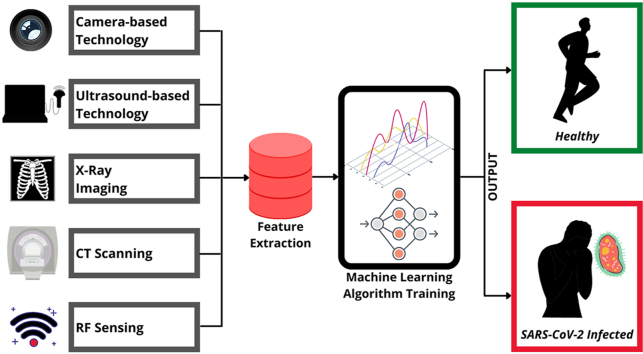

## 1 Introduction

A coronavirus is a large group of viruses that includes the Middle East respiratory syndrome coronavirus (MERS-CoV), the severe acute respiratory syndrome coronavirus (SARS-CoV), and the most recent one, SARS-CoV-2, also known as coronavirus disease 2019 (COVID-19). In early December 2019, SARS-CoV-2 occurred and eventually affected more than 250 million people globally. According to a recent figure by the World Health Organization (WHO), more than 5 million deaths from SARS-CoV-2 have been reported [1]. The distinct variants of COVID-19 are targeting lungs, consequently resulting severe damage towards breathing [[2], [3], [4], [5]]. The virus spreads through human-to-human or human-to-surface interactions and mainly triggers flu, fever, cough, ageusia, and respiratory problems [[6], [7], [8], [9], [10]]. Governments around the globe are trying to prevent the spread of the virus through constant lockdowns, which highly affect the economy and small/big businesses [[11], [12], [13]]. To stop the spread of the virus, self-quarantine is imposed on normal people in their residences, which limits their activities, whereas people affected by the virus are quarantined in hospitals until fully recovered.

New discoveries are made daily about the virus and its prevention [14,15]. However, while we are writing this paper, the genuine cause of the virus and the effects of vaccination have become significant issues [[16], [17], [18], [19], [20]]. Elderly people are highly susceptible to the effects of SARS-CoV-2, and many have already lost their lives [21,22]. This is due to the fact that the immune systems of such people are insufficient to fight against the virus. The virus spreads easily when individuals are physically close, and droplets are expelled by infected people through coughing or sneezing, which primarily endangers the life of healthcare workers [23,24]. Although the WHO has suggested infected persons and healthcare workers wear face masks and other mouth coverings, the complete effectiveness of face masks is still debatable [[25], [26], [27]].

At this stage, with the trials having ended, developed nations around the world have started vaccinations for elderly people; however, the number of cases is increasing [28]. This raises a question regarding the efficiency of the vaccine [[29], [30], [31]]. However, until a recovery occurs, governments are imposing sets of rules on people, such as social distancing and mask wearing [32,33]. Furthermore, the detection and monitoring of SARS-CoV-2 is challenging owing to certain limitations, such as shortage of equipment, facilities, and trained staff. In addition, there is a fear of exposing healthcare staff to diseased patients [[34], [35], [36]].

The United Kingdom's National Health Service and other health services around the globe are in high demand by healthcare workers as they come into contract with the virus through infected patients [[37], [38], [39]]. Non-invasive healthcare is the only possible solution to effectively reducing the spread of the virus. Technologies are rapidly developing in the current era, and non-invasive healthcare has attracted the attention of many researchers around the globe, which may ease the workload on health workers [[40], [41], [42], [43]]. Moreover, the development of healthcare technologies will significantly reduce the number of valuable clinical resources.

In this paper, we discuss and compare the state-of-art healthcare technologies and machine learning algorithms that can be effectively utilized for the detection and monitoring of COVID-19 symptoms. Camera-based technology and wearable sensors are decent solutions; however, wearable sensors may cause discomfort and risk of transferring the virus from one individual to another. On the other hand, camera-based monitoring raises privacy concerns and is limited to light and requires high computational power for further processing [[44], [45], [46]]. Non-contact wireless sensing technology is a promising solution that can effectively be used in healthcare and for the detection and monitoring of SARS-CoV-2 affected patients [[47], [48], [49]].

## 2 Intelligent healthcare technology

In recent years, enormous studies have been conducted on healthcare technologies [50,51]. These technologies are associated with either invasive or non-invasive healthcare. In terms of healthcare, non-invasive (or non-contact) technology can be used for monitoring patients without any physical contact with the body, whereas invasive (or contact) technology requires direct physical bodily contact [[52], [53], [54], [55], [56]].

**Fig. 1:**
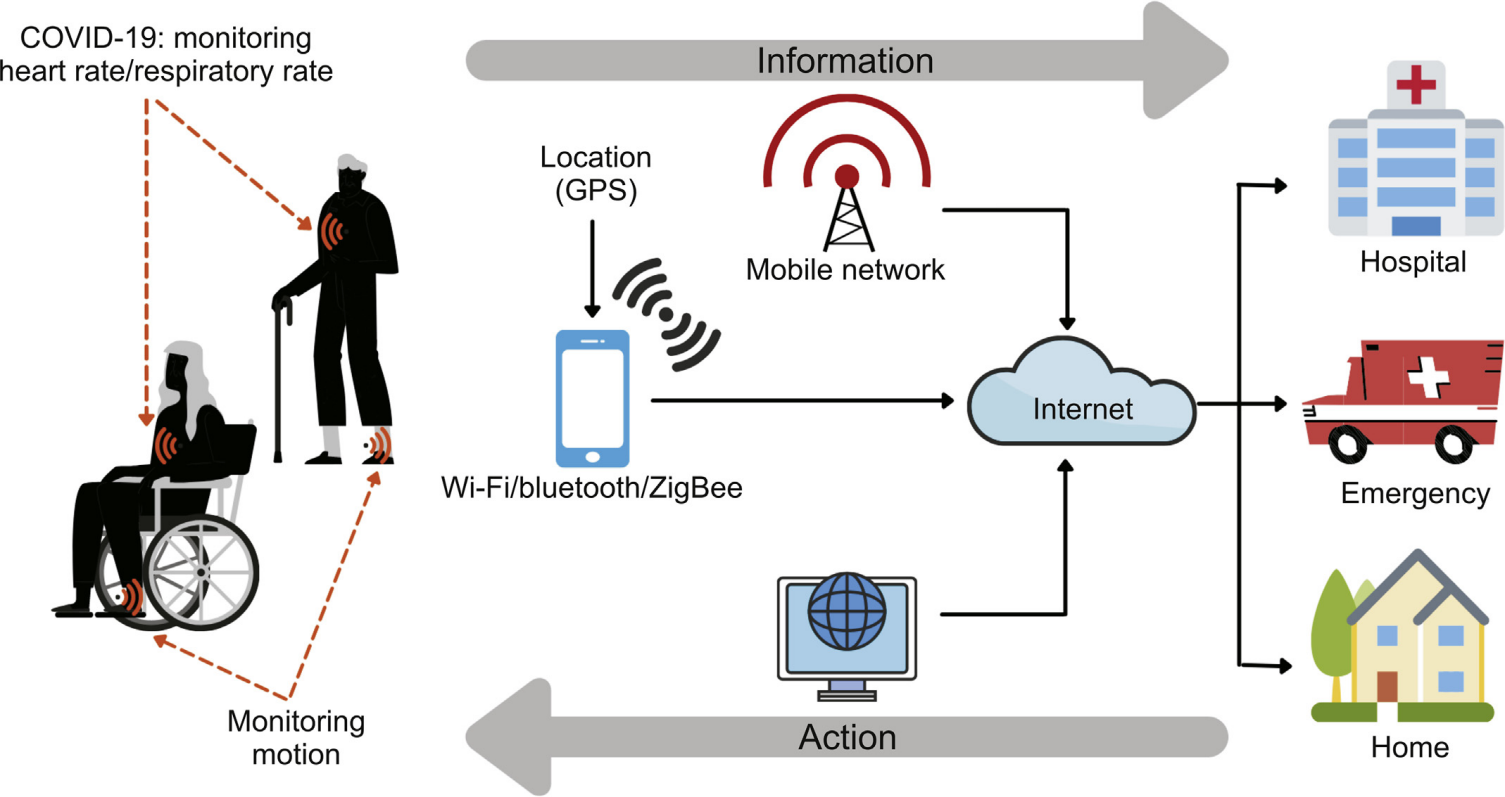
Coronavirus 2019 (COVID-19) symptom monitoring system through wireless sensing. Distinct sensors are connected to the body and information is transmitted through a gateway such as a cell phone. Actions are applied by caretakers according to the conveyed information through sensors.

In Fig. 1, an illustration of the monitoring system is shown, where wearable sensors are connected to the patient's body and data are transmitted to the care providers wirelessly. Wearable sensors gather data about a patient's physiological and movement status [[57], [58], [59], [60]]. For instance, the respiratory rate of patients with chronic obstructive pulmonary disease and the heart rate of patients with congestive heart failure are monitored using attached sensors. Currently, smart watches have the ability to monitor the heart rate and convey information in real time to the care provider [[61], [62], [63]]. All wearable devices must be in physical contact with the body to convey information. This situation causes discomfort and limitations in the case of monitoring SARS-CoV-2 affected patients because the virus can be transmitted through wearable devices. In addition, wearable devices require battery power and must be detached from the patient's body for charging. For home-based monitoring, cultural stigma associated with the use of medical devices attached to the body might occur.

To overcome the limitations of wearable sensing technology, non-contact healthcare has been proposed by researchers [[64], [65], [66]]. As the name suggests, non-contact technology requires no direct contact with the body to monitor the patient status. Owing to the COVID-19 pandemic, non-contact healthcare has recently gained immense importance. Although wearable devices can be effectively used to monitor SARS-CoV-2 affected patients, they are at the cost of potential exposure to the virus. Healthcare workers, particularly nurses, may easily be affected when attaching, changing, or removing wearable devices [67]. All possible precautions such as wearing masks or gloves, when taken by the healthcare providers, will reduce the risk of contracting the virus; nevertheless, using non-contact technology will successfully eliminate all possibilities through which the virus can be transmitted.

Healthcare technology (invasive/non-invasive) merged with artificial intelligence (AI) can significantly reduce the work pressure on hospital staff [[68], [69], [70], [71]]. For instance, radio frequency (RF) sensing technology is able to collect information from a patient's body, and the passing of this information through AI algorithms will yield valuable results without any direct involvement of hospital workers [72,73]. Remote non-contact sensing technologies integrated with smart machine learning algorithms are capable of providing correct outcomes in real time, which can easily be used by the clinician to monitor and diagnose a disease [74]. Non-contact sensing can assist healthcare staff in detecting and monitoring SARS-CoV-2 affected individuals. This will enable healthcare workers to rapidly diagnose the disease and make the right decisions on time.

**Fig. 2:**
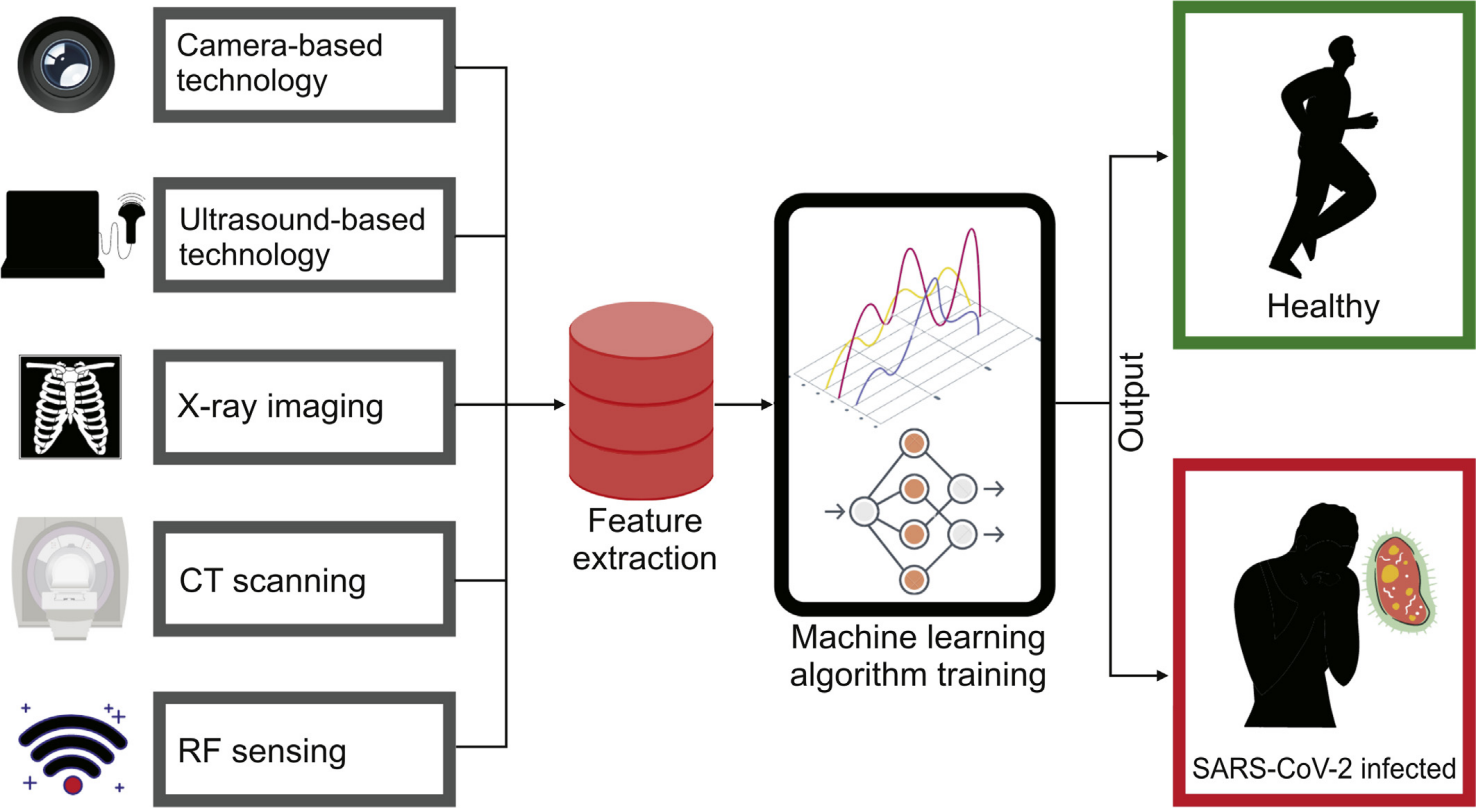
COVID-19 symptom detection and monitoring system through distinct invasive/non-invasive technologies merged with intelligent AI techniques. X-ray: X-radiation; CT: computerized tomography; RF: radio-frequency.

To fight against SARS-CoV-2, the monitoring of crucial signs is of significant importance. These signs include respiratory problems, cough, fever, and in some cases, the affected cardiovascular system [[75], [76], [77], [78]]. Shortness of breath is a vital sign when a patient experiences serious difficulty. To detect abnormal respiration, non-contact techniques can be used for monitoring diseased patients. In addition, non-contact methods can also be used to monitor the heart rate of patients suffering from the affected cardiovascular system [79,80]. All major non-contact techniques that can be effectively used for the detection and monitoring of COVID-19 symptoms are described in this paper. Moreover, Table 1 [[81], [82], [83], [84], [85], [86], [87], [88], [89], [90], [91], [92], [93]] summarizes studies in which different non-contact sensing technologies are effectively used to monitor an abnormal breathing rate, which is the primary sign of COVID-19. Fig. 2 illustrates a general system based on non-contact sensing techniques and smart AI algorithms for detecting and monitoring of COVID-19 symptoms.

**Table 1:** Summary of invasive/non-invasive technologies used to detect and monitor abnormal respiratory rate.

Technology	Data	Results	Refs.
Camera	A total of 12 male and female volunteers were recorded at distinct resolutions	100% accuracy on HD 720	[81]
Near-infrared camera	A total of 28 near-infrared videos and 11 with subject were uncovered and partially covered	99.70% and 88.95% accuracy	[82]
Smartphone camera	A total of 11 healthy subjects were recorded at distinct breathing frequencies	1.43% average median error	[83]
Thermal and depth camera	Physical activities were recorded by the home exercise bike	100% accuracy approximately	[84]
Thermal camera	A total of 41 adults and 20 children undergoing elective polysomnography were recorded	*r*=0.94 (correlation between thermal imaging and the contact method)	[85]
Ultrasound imaging	A total of 1,103 images (172 healthy, 277 pneumonia, and 654 COVID-19)	89% accuracy	[86]
Ultrasound imaging	A total of 623 videos including 99,209 ultrasound images of 70 patients	92.4% and 91.1% accuracy	[87]
X-radiation (X-ray) imaging	A total of 500 X-ray images in integration with generative adversarial networks	95.2%–97.6% accuracy	[88]
X-ray imaging	A total of 6,432 chest X-ray scan samples	97.97% accuracy	[89]
Computerized tomography (CT) scanning	A total of 150 CT images containing 53 cases of COVID-19	99.64% accuracy	[90]
CT scanning	A total of 249 CT images (COVID-19)	91.6% accuracy	[91]
Radio-frequency (RF) sensing	A total of 10 healthy humans were instructed to imitate six distinct breathing patterns	94.7% accuracy	[92]
RF sensing	Wireless data (normal, shallow, and elevated breathing)	91% accuracy	[93]

### 2.1 Camera-based technologies

**Fig. 3:**
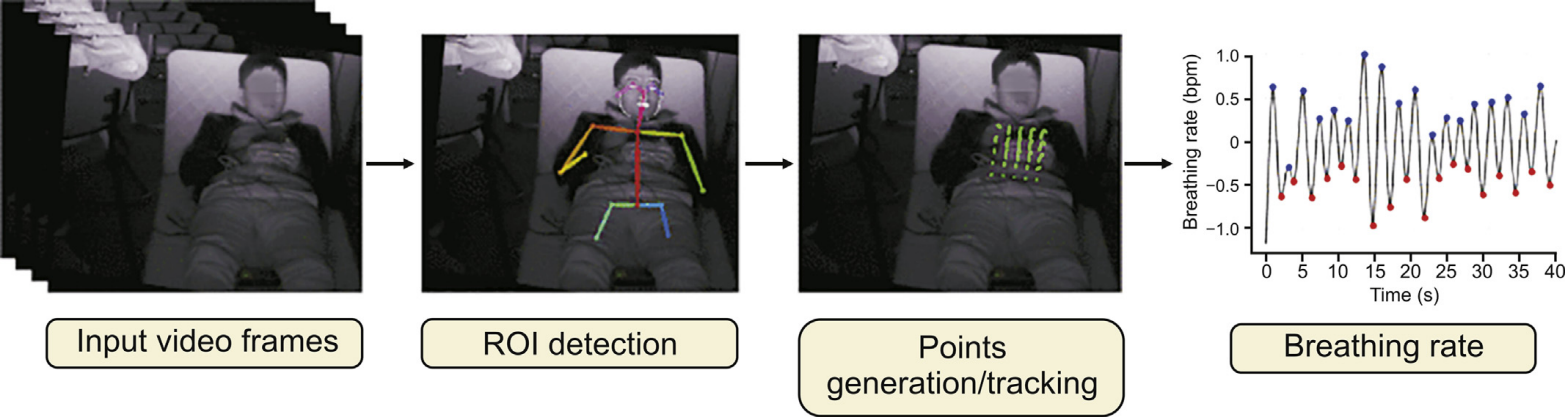
Camera-based breathing rate monitoring approach (reprinted from Ref. [82] with permission). ROI: region of interest.

Camera-based technologies can be effectively utilized [[81], [82], [83], [84], [85]]. One of the key symptoms of COVID-19 is shortness of breath (or respiratory problems), which affects chest movement. Camera-based technologies such as smartphones and thermal or depth cameras capture the video footage of the chest movements, which can then be analyzed by intelligent machine learning algorithms to detect any abnormalities in the breathing pattern (Fig. 3) [82].

### 2.2 Ultrasound-based technology

**Fig. 4:**
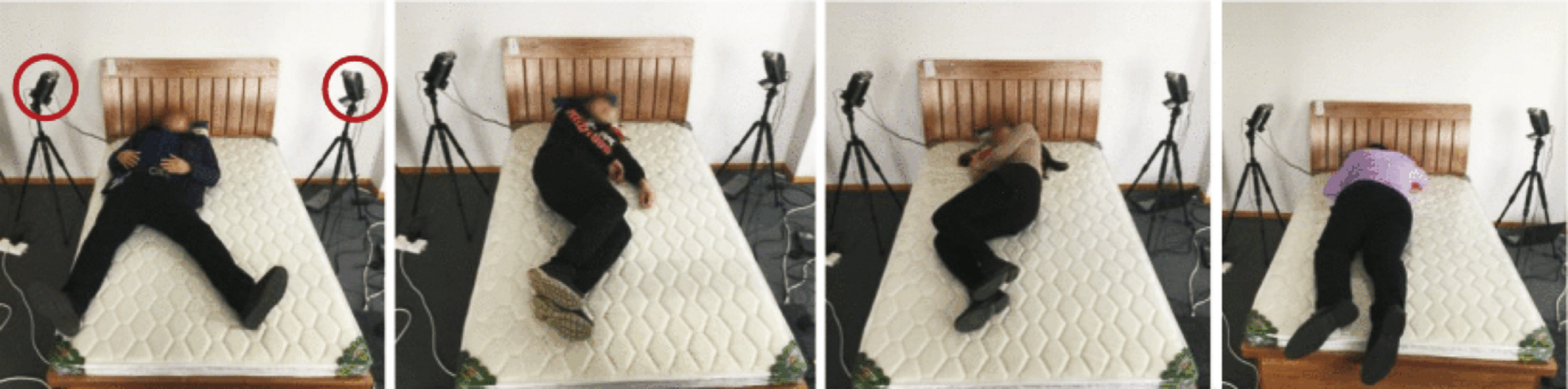
Experimental setup to monitor abnormal respiratory using ultrasound signals (reprinted from Ref. [99] with permission).

Ultrasound-based technology utilizes high-frequency sound waves to capture the body motion. To detect abnormal respiration, an ultrasound machine generates sound waves that, upon bouncing off from distinct sections of the body, generate echoes that are identified by the probe and are exploited to produce a stirring image [[94], [95], [96]]. To reduce the risk of infection from COVID-19 patients, non-contact ultrasound-based technology can be utilized by monitoring abnormal respiratory activity in the lungs (Fig. 4) [[97], [98], [99]].

### 2.3 X-radiation (X-ray) imaging

**Fig. 5:**
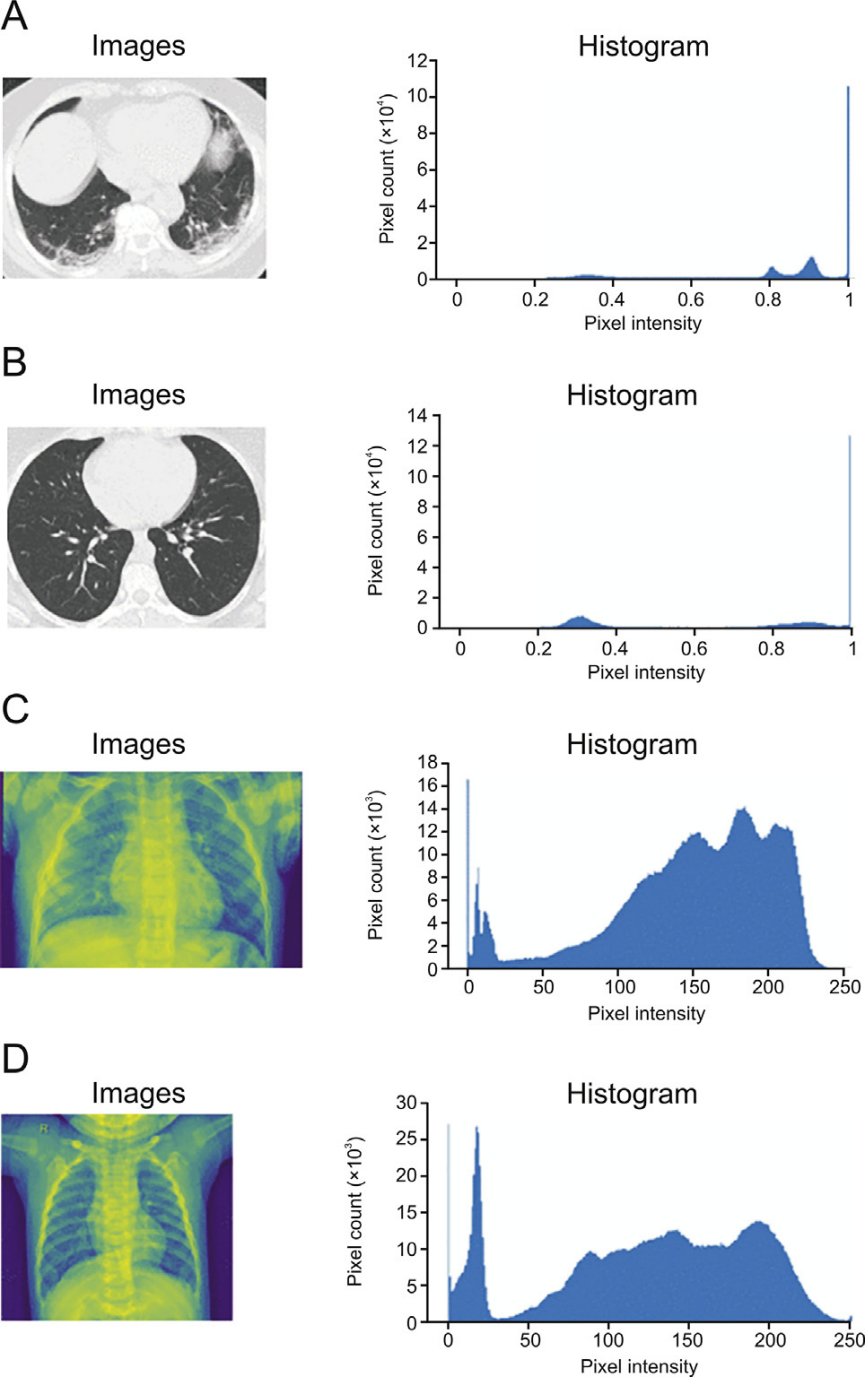
Sample images of normal person and patients with COVID-19 (left) and histograms of the images (right). CT scanning of (A) patients with COVID-19 and (B) normal person. X-ray of (C) patients with COVID-19 and (D) normal person. (Reprint from Ref. [100] with permission).

X-ray images can be used to detect and monitor the symptoms of patients with COVID-19 (Fig. 5) [100]. For instance, X-ray images of the lungs are used to detect abnormal respiration. In the past, X-ray imaging has been used to detect pneumonia. However, pneumonia and COVID-19, both as diseases, affect the respiratory system, and to detect any anomalies in the respiratory system, X-ray imaging processed through AI algorithms can be effectively used to monitor COVID-19 symptoms [[101], [102], [103]]. As limitations of X-ray imaging, it requires a professional analysis, and the equipment is costly.

### 2.4 Computerized tomography (CT) scanning

CT scanning technology involves generating X-ray imaging of a patient's chest to produce a 3D image of the lungs [104]. The final outcome of the images reveals any types of abnormalities that can eventually be utilized to detect symptoms of COVID-19 (Fig. 5) [100,[105], [106], [107]]. Research has shown that CT scanning has an exposure sensitivity of 86%–98% and can hence monitor any activity of SARS-CoV-2 infection in the lungs [108].

### 2.5 RF sensing

RF sensing primarily consists of Wi-Fi and radar-based technologies. Radar-based technology uses a frequency-modulated continuous wave to observe the Doppler effect when the whole body or part of the body moves [[109], [110], [111], [112]]. This technology can be effectively used to monitor respiratory abnormalities. Images taken by the radar system can be processed using intelligent machine learning algorithms.

**Fig. 6:**
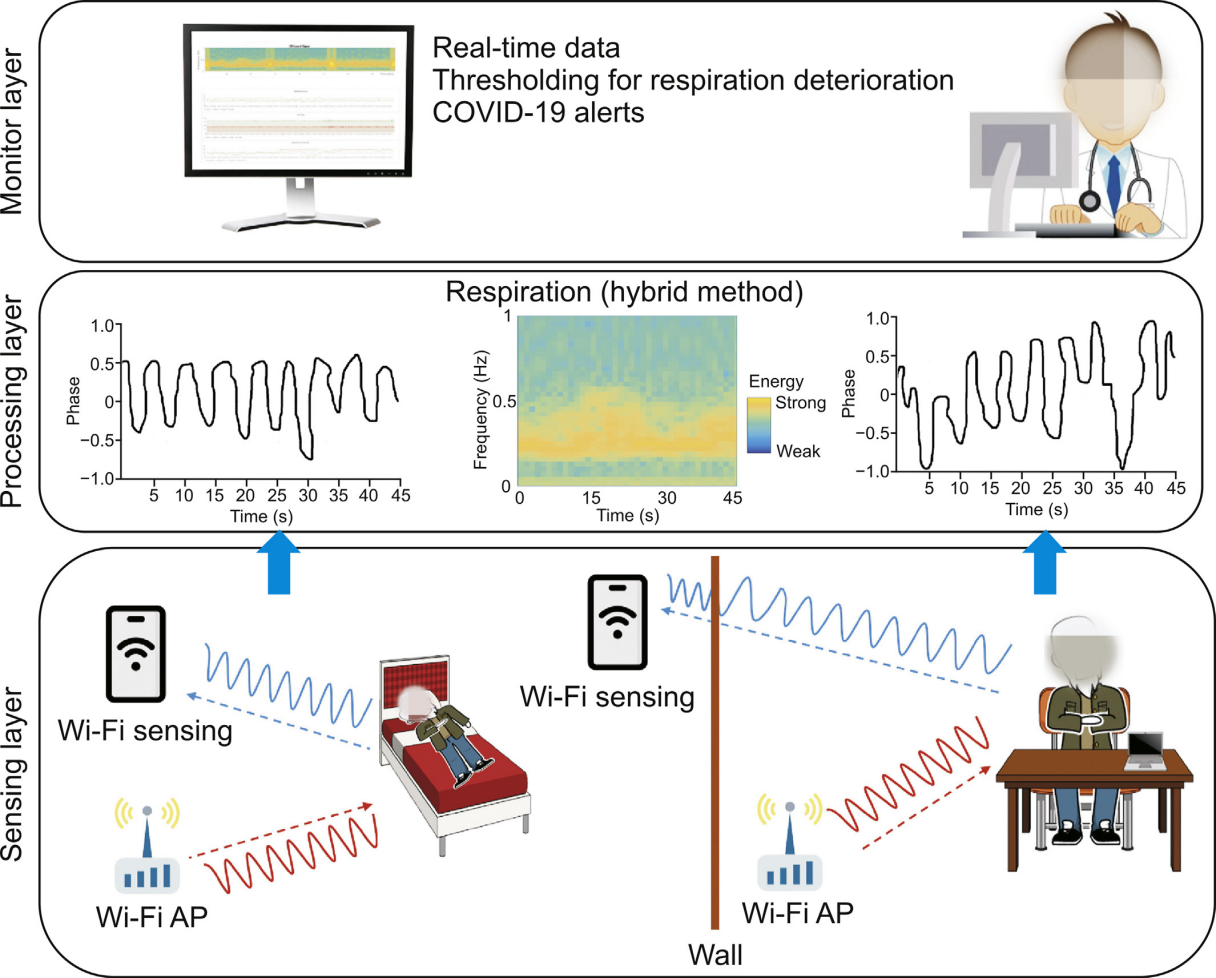
Abnormal respiratory monitoring system using Wi-Fi sensing technology (reprinted from Ref. [42] with permission).

Once trained, these algorithms can detect anomalies in images [[113], [114], [115], [116], [117][113], [114], [115], [116], [117]]. Moreover, Wi-Fi-based technology comprises wireless channel information. Wireless channel information is used to define the propagation properties of the RF signal, including the abnormalities generated by the human body parts. Wi-Fi-based sensing technology used to monitor patients with COVID-19 has opened new doors for research in terms of healthcare (Fig. 6) [42]. The received signal strength indicator obtained from Wi-Fi signals can be utilized to monitor distinct human activities.

## 3 Machine learning for detection of COVID-19 symptoms

**Fig. 7:**
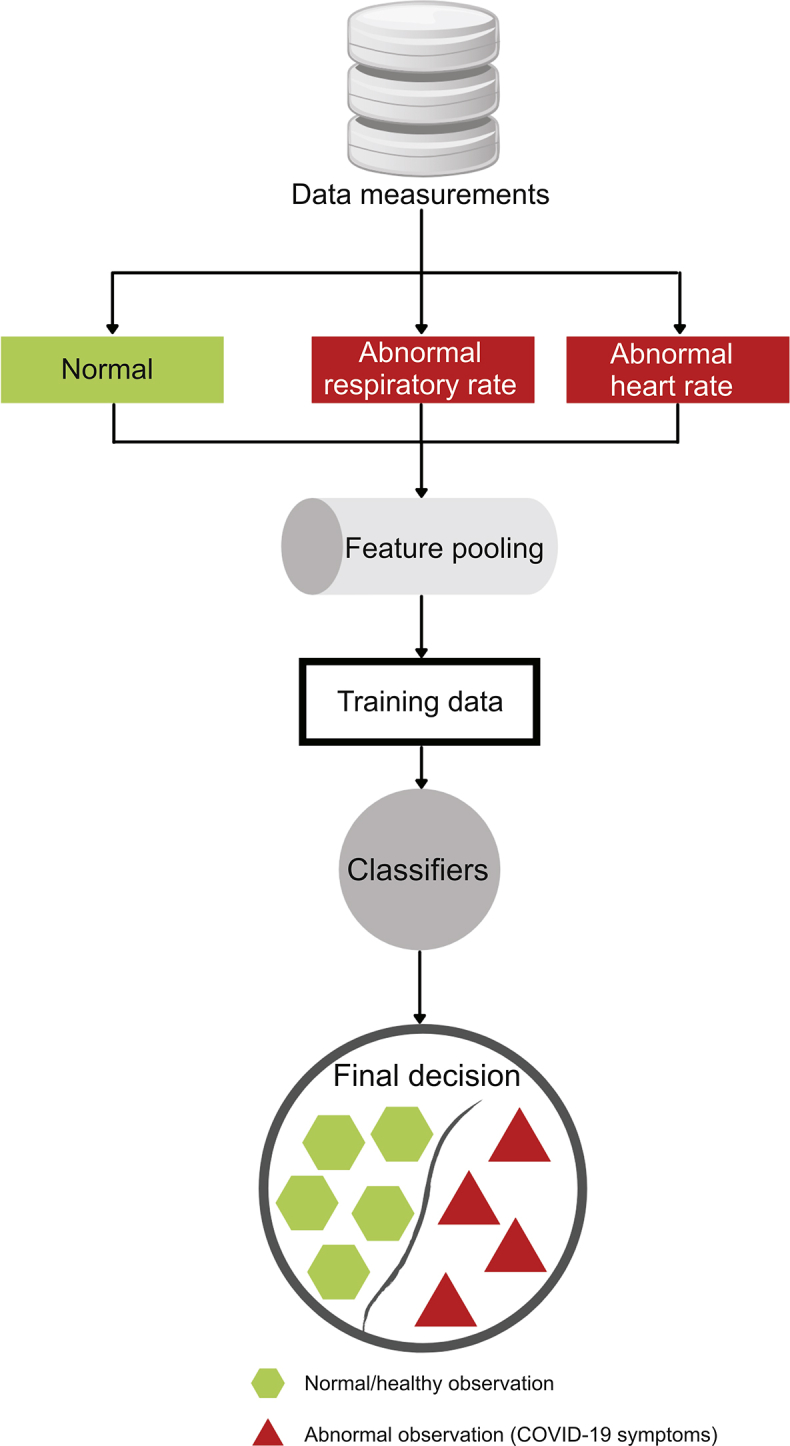
Generic framework toward COVID-19 symptom detection.

**Fig. 8:**
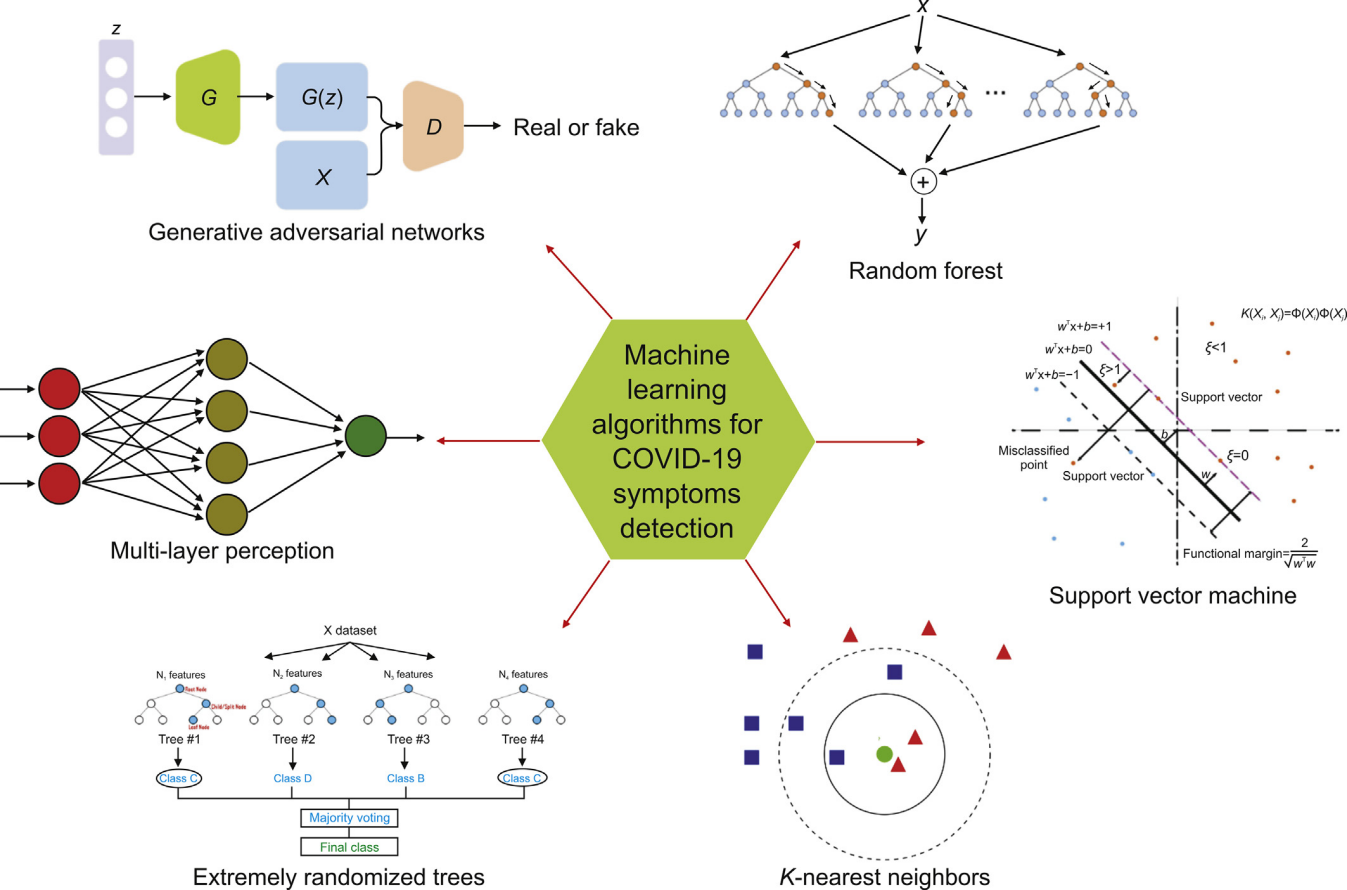
Distinct effective machine learning classifiers used to detect and monitor symptoms of COVID-19. *G*: generator; *D*: discriminatror.

In recent years, a large number of real-world applications have shifted from manual systems to automated systems based on data-driven approaches [[118], [119], [120], [121], [122]]. As the name suggests, a data-driven approach requires some amount of data be integrated with an intelligent AI algorithm. Three general steps used to describe a data-driven approach are as follows. First, data are collected for different applications. Second, the data are cleaned by extracting the important features. Third, machine learning algorithms are trained based on the features extracted from the data. In supervised machine learning, classification is a technique that categorizes the data into a distinct set of classes based on the diversity in the features [[123], [124], [125]]. In this section, some state-of-art machine learning classification algorithms, which are successfully used in modern healthcare systems, are described. In Table 2 [[126], [127], [128], [129], [130], [131], [132], [133], [134], [135], [136], [137], [138], [139], [140], [141], [142], [143], [144], [145], [146], [147], [148]], existing contributions of classification algorithms utilized for healthcare are listed. Moreover, Fig. 7 shows a general framework based on data wrangling and machine learning for abnormality classification. Feature extraction and pooling is an important step toward classification, assisting in a dimensionality reduction and thereby reducing the computational complexity. In the case of digital signal processing, the extracted features are primarily in the form of time and frequency domains, as listed in Table 3, Table 4, respectively. Furthermore, Fig. 8 reveals six distinct machine learning classification algorithms, which are described as follows.

**Table 2:** List of existing contributions in human activity monitoring and COVID-19 symptoms detection through invasive/non-invasive technology.

Detection/monitoring	Classification technique	Technology	Accuracy (%)	Refs.
Human motion	Hidden Markov Model	Wi-Fi sensing	94.2	[126]
Human motion	Support vector machine (SVM)	Wi-Fi sensing	99	[127]
Running, walking, standing, and sitting	SVM/long short-term memory	Wi-Fi sensing	95	[128]
Human presence (static/dynamic)	Naïve Bayes	Wi-Fi sensing	99	[129]
Whole body motion	Convolutional neural network	Wi-Fi sensing	90	[130]
Sitting, walking, and jogging	Auto-encoder	Wi-Fi sensing	91.1	[131]
Empty, sitting, standing, and walking	Recurrent neural network	Wi-Fi sensing	90	[132]
Sleep	K-nearest neighbors (KNN)	Wi-Fi sensing	93.88	[133]
Heart rate and respiratory rate	Dynamic time warping	Wi-Fi sensing	94	[134]
Respiration rate	Exponentially weighted moving average	Wi-Fi sensing	93.04	[135]
Walking, jogging, standing, and sitting	K-means	Radar sensing	85	[136]
Running, walking, and crawling	KNN	Radar sensing	93	[137]
Respiration rate	SVM	Software-defined radio	85	[138]
Standing up/sitting down	Random forest (RFo)	Software-defined radio	96.70	[139]
Lying, crawling, walking, and standing	KNN	Software-defined radio	85	[140]
Lying, sitting, and standing	RFo	Ultra-wideband radio	95.6	[141]
COVID-19 symptoms	RFo	Chest X-ray	97	[142]
COVID-19 symptoms	Bagging tree	X-ray and CT images	99	[143]
COVID-19 symptoms	SVM	X-ray	85.96	[144]
COVID-19 symptoms	RFo	Blood test	97	[145]
COVID-19 symptoms	RFo	Blood test	86	[146]
COVID-19 symptoms	Naïve Bayes	Textual clinical reports	96.2	[147]
COVID-19 symptoms	ResNet-50	Coughs recorded on smartphone	95.01	[148]

**Table 3:** Time-domain features.

Title	Expression
Mean	$\frac{1}{N}\sum_{i=1}^{N}x_{i}$
Min	*Min (X*_*i*_*)*
Max	*Max (X*_*i*_*)*
Variance	$\sum_{i=1}^{N}{\left(x_{i}-u_{x}\right)}^{2}$
Root mean square	$\sqrt{\frac{1}{N}\sum_{i=1}^{N}x_{i}^{2}}$
Kurtosis	$\frac{1}{N}{\sum_{i=1}^{N}\left(\frac{x_{i}-u_{x}}{\sigma }\right)}^{4}$
Skewness	$\frac{1}{N}{\sum_{i=1}^{N}\left(\frac{x_{i}-u_{x}}{\sigma }\right)}^{3}$
Range	*x*_max_ − *x*_min_
Interquartile range	*Q*_*3*_*-Q*_*1*_
Standard deviation	$\sqrt{\frac{1}{N}{\sum_{i=1}^{N}\left(x_{i}-\mu \right)}^{2}}$

**Table 4:** Frequency-domain features.

Title	Expression
Signal energy	$\sum_{n=-N}^{N}{\left|p\left(d\right)\right|}^{2}$
Spectrum entropy	$\sum_{i=-N}^{N}p\left(d\right)\ln\left(p\left(d\right)\right)$
Fast Fourier transform	$\sum_{n=-N}^{N}x\left(n\right)e^{-j\frac{2\pi }{N}nd}$
Frequency peak	*Max* (*FFT*(*d*))
Spectral probability	$\frac{FFT{\left(d\right)}^{2}}{\sum_{i=-N}^{N}FFT{\left(i\right)}^{2}}$

### 3.1 Generative adversarial networks (GANs)

GANs have recently been employed for numerous healthcare-related applications, particularly SARS-CoV-2 [[149], [150], [151]]. A GAN is an approach based on a deep learning technique that consists of two models: a generator and a discriminator [152]. A GAN is used primarily to discover and learn hidden patterns in the data in such a way that the model can be utilized to create new replicated data samples. By treating the problem as supervised, a GAN is an intelligent method for training a generator model. Fundamentally, the generator model is trained to produce new data samples, and the discriminator model can classify those samples as either real or fake. Both models are trained in a zero-sum game process until the discriminator model is deceived. The GAN is extremely effective for problems in which the number of training data samples is insufficient [153]. For instance, when dealing with multi-class classification problems, there might be unbalanced classes, and hence, the GAN can be utilized to generate new samples and balance the classes.

The step-by-step training process of a GAN is described as follows. First, noise is added to the generator (*G*) from a random distribution to generate the fake *y* (label *z*=0) → (*y, z*) input label pair. Second, the real pair *y* (label *z*=1) and fake pair are alternatively supplied to the discriminator (*D*). Third, the *D* as a neural network binary classifier calculates the final loss *D*_*loss*_ by combining the loss for both the real *y* and fake *y*. Fourth, because each model has distinct objective functions, the *G* calculates the loss from its noise as *G*_*loss*_. Fifth, both models *G* and *D* learn from the loss and thus adjust the parameters. Sixth, the optimization algorithm is applied. Seventh, step 6 is repeated for a specific number of epochs.

### 3.2 Random forest (RFo)

RFo is an ensemble learning-based algorithm that is primarily used for classification and regression tasks. During RFo training, several decision trees are generated, and a final class is produced, which is the mean prediction of individual trees. For classification tasks, the RFo can classify the data using the rules generated for the test features and each randomly formulated decision tree. First, for each targeted value, the numbers of votes created by the decision trees are calculated. Second, for the final RFo result, the majority voted prediction target is considered [[154], [155], [156]].

### 3.3 Multi-layer perceptron (MLP)

The MLP is a class of feedforward artificial neural networks and comprises at least three layers of nodes, that is, input, hidden, and output. A supervised machine learning technique called back-propagation is used by the MLP for the training process. In addition, each neuron uses a non-linear activation function, except for the input neurons. Furthermore, the number of neurons or the number of hidden layers in the MLP is determined during the training process. Problems of over-fitting or under-fitting of the model may occur if the optimal numbers of neurons or hidden layers are not achieved during training [[157], [158], [159]].

### 3.4 Support vector machine (SVM)

The SVM algorithm works based on the concept of statistical learning theory, and in multi-class classification problems, an SVM is one of the best performing machine learning algorithms. Many researchers have utilized and suggested SVMs for distinct real-life applications such as a system fault diagnosis or abnormality detection and the monitoring of patients [[160], [161], [162], [163]]. The SVM algorithm generates a hyperplane or linear line as a decision boundary to separate different types of data points for classification tasks. The data points close to the hyperplane imparting structure of the hyperplane are known as support vectors. An optimal hyperplane can be expressed as

(1) $$w^{T}x+b=0$$

where *w* is the vector of the weights, *b* represents the bias, and *x* is an input vector. The equations for the support vectors of each class are as follows:

(2) $$\begin{array}{ll} w^{T}x+b=+1, & for\;d_{i}=+1\\ w^{T}x+b=-1, & for\;d_{i}=-1 \end{array}$$

where *d*_*i*_ corresponds to the respective class, such as +1 for class A and −1 for class B.

### 3.5 Extremely randomized trees (ERTs)

An ERT is an ensemble learning-based algorithm that has recently attracted the attention of many researchers. An ERT is a modified form of an RFo algorithm, which can be used for classification and regression tasks. As the name suggests, an ERT is an extremely random training technique based on decision trees. The random nature of an ERT makes it perfectly suitable for the training of a large number of datasets, where each feature in the data is highly repetitive and hence makes the training process computationally expensive. Many studies have recently revealed the efficacy of an ERT and how it can be utilized for a large number of real-world applications, including healthcare [[164], [165], [166], [167]].

### 3.6 K-nearest neighbors (KNNs)

A KNN is a commonly used algorithm, particularly for classification tasks, when the size of the data is not large. Based on the nearest neighbor algorithm, the KNN selects *k* nearest neighbors instead of selecting only the first nearest neighbor, while *k* is the number of nearest neighbors to an object identified by the model. Some of the important parameters to be considered while training the KNN classifier are the number of neighbors, distance metric, leaf size, and weights [[168], [169], [170]].

## 4 Limitations

In this paper, we discussed the details of modern healthcare techniques merged with AI and how they can be used effectively to prevent the spread of SARS-CoV-2 through the timely detection and monitoring of symptoms. However, there are still limitations associated with non-contact sensing technology, which require further exploration.

### 4.1 Privacy

Although RF sensing is a promising technology when it comes to monitoring SARS-CoV-2 affected individuals, nevertheless, it may cause privacy and security concerns. For instance, Wi-Fi sensing can intervene with other potential users, and a hacker may be able to monitor the activities of a targeted subject. In some cases, even false alarms can be generated. In addition, camera-based technology may cause discomfort and privacy concerns for the subject being monitored.

### 4.2 Location

In RF sensing-based systems, the subject's location and orientation have vital consequences on the performance. The disparities between location and orientation information may cause distinct variations in the wireless channel state information. Although some studies have examined different locations and orientations, there is still a need for further research to overcome this challenge.

### 4.3 Environmental impact

In real-time monitoring systems, the environment plays a vital role because it continuously interacts with RF-based technologies such as Wi-Fi sensing. Environments can cause a disruption in the form of moving doors, windows, furniture, and electronic devices. These disruptions in the environment can significantly affect the performance of wireless channels. Even though supervised machine learning classifiers can detect human activities with maximum accuracy, they cannot adopt a new environment by themselves. It is essential to utilize techniques such as reinforcement learning, which can be altered with changes in the environment.

### 4.4 Multiple subjects

To detect and monitor SARS-CoV-2 affected individuals using RF sensing technologies, most of the current studies have conducted experiments with either a single or limited number of subjects. Although RF sensing can effectively monitor a single subject once quarantined, it can be difficult to monitor several subjects simultaneously. Disruptions can be caused by the movement of several subjects.

### 4.5 Experiment constraints

To conduct any type of experiments, the accuracy of the data acquisition part must be significant. In health-related experiments, it is complicated to conduct tests on real patients. The data used by the researchers to conduct experiments are primarily generated by volunteers and not real subjects. This causes certain limitations because an actually affected patient might reveal distinct actions in comparison to the volunteers. Moreover, with the new variants of SARS-CoV-2, the symptoms may vary from subject to subject.

## 5 Future directions

In this paper, we discussed all the possible technologies (contact/non-contact) that can be effectively used to detect and monitor the symptoms of COVID-19. The most promising technology among all is based on RF sensing, which does not require any contact with the subject to be monitored. However, there are still some challenges associated with contact and non-contact techniques that need to be addressed in the future.

1) Camera-based technologies can detect the symptoms of COVID-19 by monitoring irregular respiratory rates. However, there are certain limitations associated with this approach, such as light dependency, privacy concerns, and a high computational power. In addition, camera-based technologies cannot categorically determine SARS-CoV-2 as a cause for individuals. In the future, it is recommended to design and develop a real-time model based on extensive experiments that can overcome the limitations caused by camera-based technology.
2) Ultrasound-based technology can be made portable by utilizing mobile devices. To identify the symptoms of COVID-19, ultrasound-based images of the lungs can be inspected using machine learning classifiers. For a future study, it is recommended to establish mobile device applications that can capture a lung ultrasound. This technique enables users to utilize mobile devices to detect and monitor abnormal symptoms.
3) X-ray and CT scanning technologies have revealed fine results for the detection of COVID-19. However, the major limitations of these technologies are portability and the risk of exposure to radiation. Thus, they cannot be widely used for screening patients with COVID-19. Although both of these technologies are highly precise, they still require the subject to be in a certain location where X-ray and CT scan technologies are available. The advantage of these technologies is that they provide high-resolution images, and once trained by intelligent machine learning algorithms, they will be able to produce highly accurate results. It is recommended to conduct comprehensive experiments with large data sizes, which can eventually result in a more robust model. This will allow for the accurate and rapid detection of COVID-19 symptoms.
4) RF-based systems such as Wi-Fi sensing are inexpensive and reliable techniques for detecting, diagnosing, and monitoring the symptoms of COVID-19, such as abnormal respiratory and heart rates. The Wi-Fi-based sensing technology can be implemented using existing Wi-Fi devices available within hospitals and homes. RF-based technology has the potential to monitor subjects without any external human interaction, which is needed in the case of COVID-19. It is highly recommended to utilize RF sensing-based technologies in future research.
5) Most of the current research related to non-invasive healthcare for COVID-19 is based on supervised and semi-supervised machine learning techniques. With these techniques, the majority of research has shown promising results. Nevertheless, to develop an automated system with adaptability to the environment, techniques such as reinforcement learning must be considered in the future.
6) It is recommended to explore and conduct experiments on genuine multi-patient data in a real environment, rather than using data from healthy volunteers in the laboratory.

## 6 Conclusions

At the current stage, more than five million fatalities have been reported owing to SARS-CoV-2, and with the recently discovered novel SARS-CoV-2 Delta and Omicron variants, the mortality rate is expected to increase because existing vaccinations are not entirely effective against these variants. Wireless or non-contact technology is important, particularly during the COVID-19 pandemic, as they demand the least amount of participation from infected individuals and healthcare workers. In this paper, we presented a detailed review of COVID-19 and its prevention through contact/non-contact healthcare technologies, such as radio-frequency sensing. The non-contact technologies have the ability to terminate human-to-human interaction, which is necessary to contain SARS-CoV-2 infections. Radio-frequency-based systems such as software-defined radio, radar, and Wi-Fi sensing are promising non-contact solutions that can be effectively utilized to detect, diagnose, and monitor COVID-19 symptoms, for example, unusual respiration in the form of shortness of breath. Moreover, we discussed state-of-art machine learning algorithms, including generative adversarial networks, and how they are exploited in healthcare-related applications. In addition, this study highlighted the limitations associated with non-contact technologies as well as potential future research directions.

## CRediT author statement

**Umer Saeed:** Conceptualization, Methodology, Software, Validation, Formal analysis, Investigation, Data curation, Writing - Original draft preparation, Reviewing and Editing, Visualization; **Syed Yaseen Shah:** Validation, Formal analysis, Investigation; **Jawad Ahmad:** Validation, Formal analysis, Investigation; **Muhammad Ali Imran:** Validation, Formal analysis, Investigation; **Qammer H. Abbasi:** Conceptualization, Formal analysis, Resources; **Syed Aziz Shah:** Conceptualization, Methodology, Formal analysis, Investigation, Resources, Supervision.

## Declaration of competing interest

The authors declare that there are no conflicts of interest.

## References

[1] Report on coronavirus by World Health Organization (WHO). https://COVID19.who.int.

[2] Hellewell J., Abbott S., Gimma A. (2020). Feasibility of controlling COVID-19 outbreaks by isolation of cases and contacts. Lancet Global Health.

[3] Jiang S., Xia S., Ying T. (2020). A novel coronavirus (2019-ncov) causing pneumonia-associated respiratory syndrome. Cell. Mol. Immunol..

[4] Khan M.-A., Atangana A. (2020). Modeling the dynamics of novel coronavirus (2019-ncov) with fractional derivative. Alex. Eng. J..

[5] Nishiura H., Linton N.M., Akhmetzhanov A.R. (2020). Initial cluster of novel coronavirus (2019-ncov) infections in Wuhan, China is consistent with substantial human-to-human transmission. J. Clin. Med..

[6] Poyiadji N., Shahin G., Noujaim D. (2020). COVID-19–associated acute hemorrhagic necrotizing encephalopathy: imaging features. Radiology.

[7] Xu Z., Shi L., Wang Y. (2020). Pathological findings of COVID-19 associated with acute respiratory distress syndrome. Lancet Respir. Med..

[8] Singhal T. (2020). A review of coronavirus disease-2019 (COVID-19). Indian J. Pediatr..

[9] Pan L., Mu M., Yang P. (2020). Clinical characteristics of COVID-19 patients with digestive symptoms in Hubei, China: a descriptive, cross-sectional, multicenter study. Am. J. Gastroenterol..

[10] Dawson P., Rabold E.-M., Laws R.-L. (2021). Loss of taste and smell as distinguishing symptoms of coronavirus disease 2019. Clin. Infect. Dis..

[11] World Health Organization (19 March 2020). https://apps.who.int/iris/bitstream/handle/10665/331497/WHO-2019-nCoV-IHR_Quarantine-2020.2-eng.pdf?sequence=1&isAllowed=y.

[12] National Bureau of Economic Research, How are small businesses adjusting to COVID-19? Early evidence from a survey. https://www.nber.org/papers/w26989. (Accessed 15 July 2021).

[13] LSE Business Review, How is Covid-19 affecting businesses in the UK? https://blogs.lse.ac.uk/businessreview/2020/05/07/how-is-covid-19-affecting-businesses-in-the-uk/. (Accessed 25 July 2021).

[14] Singh R.-P., Javaid M., Haleem A. (2020). Internet of things (iot) applications to fight against COVID-19 pandemic. Diabet. Metab. Syndr. Clin. Res. Rev..

[15] Haleem A., Javaid M., Vaishya R. (2020). Effects of COVID-19 pandemic in daily life. Curr. Med. Res. Pract..

[16] Cai J., Sun W., Huang J. (2020). Indirect virus transmission in cluster of COVID-19 cases, Wenzhou, China, 2020. Emerg. Infect. Dis..

[17] Dong D., Tang Z., Wang S. (2021). The role of imaging in the detection and management of COVID-19: a review. IEEE Rev. Biomed. Eng..

[18] Lindsley A.-W., Schwartz J.-T., Rothenberg M.E. (2020). Eosinophil responses during COVID-19 infections and coronavirus vaccination. J. Allergy Clin. Immunol..

[19] Loomba S., de Figueiredo A., Piatek S.-J. (2021). Measuring the impact of COVID-19 vaccine misinformation on vaccination intent in the UK and USA. Nat. Hum. Behav..

[20] Mazereel V., van Assche K., Detraux J. (2021). COVID-19 vaccination for people with severe mental illness: why, what, and how?. Lancet Psychiatr..

[21] Daoust J.-F. (2020). Elderly people and responses to COVID-19 in 27 countries. PLoS One.

[22] Kadambari S., Klenerman P., Pollard A.J. (2020). Why the elderly appear to be more severely affected by COVID-19: the potential role of immunosenescence and CMV. Rev. Med. Virol..

[23] Zhang W.-R., Wang K., Yin L. (2020). Mental health and psychosocial problems of medical health workers during the COVID-19 epidemic in China. Psychother. Psychosom..

[24] Haleem A., Javaid M., Vaishya R. (2020). Areas of academic research with the impact of COVID-19. Am. J. Emerg. Med..

[25] Feng S., Shen C., Xia N. (2020). Rational use of face masks in the COVID-19 pandemic. Lancet Respir. Med..

[26] Howard J., Huang A., Li Z. (2021). An evidence review of face masks against COVID-19. Proc. Natl. Acad. Sci. U S A.

[27] Bakhit M., Krzyzaniak N., Scott A.-M. (2021). Downsides of face masks and possible mitigation strategies: a systematic review and meta-analysis. BMJ Open.

[28] Kim J.-H., Marks F., Clemens J.-D. (2021). Looking beyond COVID-19 vaccine phase 3 trials. Nat. Med..

[29] Wadman M. (2020). Public needs to prep for vaccine side effects. Science.

[30] Mahase E. (2020). COVID-19: vaccine candidate may be more than 90% effective, interim results indicate. BMJ Clin. Res. Ed..

[31] van Riel D., de Wit E. (2020). Next-generation vaccine platforms for COVID-19. Nat. Mater..

[32] Salathé M., Althaus C.L., Neher R. (2020). COVID-19 epidemic in Switzerland: on the importance of testing, contact tracing and isolation. Swiss Med. Wkly..

[33] Lewnard J.-A., Lo N.-C. (2020). Scientific and ethical basis for social-distancing interventions against COVID-19. Lancet Infect. Dis..

[34] Brandstetter S., Roth S., Harner S. (2020). Symptoms and immunoglobulin development in hospital staff exposed to a SARS-CoV-2 outbreak, Pediatr. Allerg. Immunol. (Leipz.).

[35] Fill Malfertheiner S., Brandstetter S., Roth S. (2020). Immune response to SARS-CoV-2 in health care workers following a COVID-19 outbreak: a prospective longitudinal study. J. Clin. Virol..

[36] Buselli R., Corsi M., Baldanzi S. (2020). Professional quality of life and mental health outcomes among health care workers exposed to SARS-CoV-2 (COVID-19). Int. J. Environ. Res. Publ. Health.

[37] Willan J., King A.-J., Jeffery K. (2020). Challenges for NHS hospitals during COVID-19 epidemic. BMJ.

[38] Pandit J. (2020). Demand–capacity modelling and COVID-19 disease: identifying themes for future NHS planning. Anaesthesia.

[39] Manzano García G., Ayala Calvo J.-C. (2021). The threat of COVID-19 and its influence on nursing staff burnout. J. Adv. Nurs..

[40] Yang X., Ren X., Chen M. (2020). Human posture recognition in intelligent healthcare. J. Phys.: Conf. Ser..

[41] Abbasi Q.-H., Ur-Rehman M., Qaraqe K. (2016). Advances in Body-Centric Wireless Communication: Applications and State-Of-The-Art.

[42] Li F., Valero M., Shahriar H. (2021). Wi-COVID: a COVID-19 symptom detection and patient monitoring framework using WIFI. Smart Health (Amst).

[43] Kapoor A., Guha S., Kanti Das M. (2020). Digital healthcare: the only solution for better healthcare during COVID-19 pandemic?. Indian Heart J..

[44] Liu J., Teng G., Hong F. (2020). Human activity sensing with wireless signals: a survey. Sensors.

[45] Ma J., Wang H., Zhang D. (2016). 2016 International IEEE Conferences on Ubiquitous Intelligence & Computing, Advanced and Trusted Computing, Scalable Computing and Communications, Cloud and Big Data Computing, Internet of People, and Smart World Congress (UIC/ATC/ScalCom/CBDCom/IoP/SmartWorld), July 18-21, 2016, Toulouse.

[46] Naudé W. (2020). Artificial intelligence vs COVID-19: limitations, constraints and pitfalls. AI Soc..

[47] Massaroni C., Nicolò A., Schena E. (2020). Remote respiratory monitoring in the time of COVID-19. Front. Physiol..

[48] Elagan S., Abdelwahab S.-F., Zanaty E. (2021). Remote diagnostic and detection of coronavirus disease (COVID-19) system based on intelligent healthcare and Internet of Things. Results Phys..

[49] Tsai C.-Y., Chang N.-C., Fang H.-C. (2020). A novel noncontact self-injection-locked radar for vital sign sensing and body movement monitoring in COVID-19 isolation ward. J. Med. Syst..

[50] Haleem A., Javaid M. (2020). Medical 4.0 and its role in healthcare during COVID-19 pandemic: a review. J. Ind. Intg. Mgmt..

[51] Javaid M., Haleem A., Singh R.-P. (2020). Industry 5.0: potential applications in COVID-19. J. Ind. Intg. Mgmt..

[52] Kahankova R., Martinek R., Jaros R. (2019). A review of signal processing techniques for non-invasive fetal electrocardiography. IEEE Rev. Biomed. Eng..

[53] Ding X., Clifton D., Ji N. (2020). Wearable sensing and telehealth technology with potential applications in the coronavirus pandemic. IEEE Rev. Biomed. Eng..

[54] Ahmadzadeh S., Luo J., Wiffen R. (2022). Review on biomedical sensors, technologies, and algorithms for diagnosis of sleep disordered breathing: comprehensive survey. IEEE Rev. Biomed. Eng..

[55] Castera L. (2020). Non-invasive tests for liver fibrosis in NAFLD: creating pathways between primary healthcare and liver clinics. Liver Int..

[56] Winck J.-C., Ambrosino N. (2020). COVID-19 pandemic and non-invasive respiratory management: every Goliath needs a David. An evidence-based evaluation of problems. Pulmonology.

[57] Yang G., Pang G., Pang Z. (2019). Non-invasive flexible and stretchable wearable sensors with nano-based enhancement for chronic disease care. IEEE Rev. Biomed. Eng..

[58] Ali F., El-Sappagh S., Islam S.-R. (2021). An intelligent healthcare monitoring framework using wearable sensors and social networking data. Future Generat. Comput. Syst..

[59] Rucco R., Sorriso A., Liparoti M. (2018). Type and location of wearable sensors for monitoring falls during static and dynamic tasks in healthy elderly: a review. Sensors.

[60] Moin A., Zhou A., Rahimi A. (2021). A wearable biosensing system with in-sensor adaptive machine learning for hand gesture recognition. Nat. Electron..

[61] Wasserlauf J., You C., Patel R. (2019). Smartwatch performance for the detection and quantification of atrial fibrillation, Circ. Arrhythmia Electrophysiol.

[62] Hoilett O.-S., Twibell A.M., Srivastava R. (2018). 2018 40th Annual International Conference of the IEEE Engineering in Medicine and Biology Society (EMBC), July 18−21, 2018.

[63] Irawan H.C., Juhana T. (2017).

[64] Taylor W., Abbasi Q.-H., Dashtipour K. (2020). A review of the state of the art in non-contact sensing for COVID-19. Sensors.

[65] Khan M.-B., Zhang Z., Li L. (2020). A systematic review of non-contact sensing for developing a platform to contain COVID-19. Micromachines.

[66] Avdeev S.-N., Yaroshetskiy A.-I., Tsareva N.A. (2021). Noninvasive ventilation for acute hypoxemic respiratory failure in patients with COVID-19. Am. J. Emerg. Med..

[67] Wang Y.-X., Guo H.-T., Du X.-W. (2020). Factors associated with post-traumatic stress disorder of nurses exposed to coronavirus disease 2019 in China. Medicine.

[68] Javaid M., Khan I.-H. (2021). Internet of Things (IoT) enabled healthcare helps to take the challenges of COVID-19 pandemic. J. Oral Biol. Craniofacial Res..

[69] Javaid M., Haleem A., Vaishya R. (2020). Industry 4.0 technologies and their applications in fighting COVID-19 pandemic. Diabet. Metab. Syndr.: Clin. Res. Rev..

[70] Wadali J.-S., Khosla P.-K. (2021).

[71] Rizwan A., Zoha A., Mabrouk I. (2020). A review on the state of the art in atrial fibrillation detection enabled by machine learning. IEEE Rev. Biomed. Eng..

[72] Shah S.-A., Abbas H., Imran M.-A. (2021). RF sensing for healthcare application. Backscattering and RF Sensing for Future Wireless Communication.

[73] Kazim J.U.R., Cui T.J., Zoha A. (2020). Wireless on walls: revolutionizing the future of health care. IEEE Antennas Propag. Mag..

[74] Alimadadi A., Aryal S., Manandhar I. (2020). Artificial intelligence and machine learning to fight COVID-19. Physiol. Genom..

[75] Fan D., Ren A., Zhao N. (2018). Breathing rhythm analysis in body centric networks. IEEE Acc..

[76] Marini J.-J., Gattinoni L. (2020). Management of COVID-19 respiratory distress. JAMA.

[77] Zheng Y.-Y., Ma Y.-T., Zhang J.-Y. (2020). COVID-19 and the cardiovascular system. Nat. Rev. Cardiol..

[78] He J., Guo Y., Mao R. (2021). Proportion of asymptomatic coronavirus disease 2019: a systematic review and meta-analysis. J. Med. Virol..

[79] Kunutsor S.-K., Laukkanen J.-A. (2020). Cardiovascular complications in COVID-19: a systematic review and meta-analysis. J. Infect..

[80] Li C., Lubecke V.-M., Boric-Lubecke O. (2013). A review on recent advances in Doppler radar sensors for noncontact healthcare monitoring. IEEE Trans. Microw. Theor. Tech..

[81] Massaroni C., Lopes D.S., Lo Presti D. (2018). Contactless monitoring of breathing patterns and respiratory rate at the pit of the neck: a single camera approach. J. Sensor.

[82] Queiroz L., Oliveira H., Yanushkevich S. (2020). 2020 IEEE International Conference on Systems, Man, and Cybernetics (SMC).

[83] Nam Y., Kong Y., Reyes B. (2016). Monitoring of heart and breathing rates using dual cameras on a smartphone. PLoS One.

[84] Procházka A., Charvátová H., Vyšata O. (2017). Breathing analysis using thermal and depth imaging camera video records. Sensors.

[85] Elphick H.-E., Alkali A.-H., Kingshott R.-K. (2019). Exploratory study to evaluate respiratory rate using a thermal imaging camera. Respiration.

[86] Born J., Brandle G., Cossio M. (2020). arXiv..

[87] Tsai C.-H., van der Burgt J., Vukovic D. (2021). Automatic deep learning based pleural effusion classification in lung ultrasound images for respiratory pathology diagnosis. Phys. Med..

[88] Rasheed J., Hameed A.A., Djeddi C. (2021). A machine learning-based framework for diagnosis of COVID-19 from chest X-ray images. Interdiscipl. Sci. Comput. Life Sci..

[89] Jain R., Gupta M., Taneja S. (2021). Deep learning-based detection and analysis of COVID-19 on chest X-ray images. Appl. Intell..

[90] Barstugan M., Ozkaya U., Ozturk S. (2020). arXiv..

[91] Shan F., Gao Y., Wang J. (2020). arXiv..

[92] Zhao H., Hong H., Miao D. (2018). A noncontact breathing disorder recognition system using 2.4-GHz digital-IF Doppler radar. IEEE J. Biomed. Heal. Informatics.

[93] Ashleibta A.-M., Abbasi Q.-H., Shah S.-A. (2020). Non-invasive RF sensing for detecting breathing abnormalities using software defined radios. IEEE Sensor. J..

[94] Powles A.-E., Martin D.-J., Wells I.-T. (2018). Physics of ultrasound. Anaesth. Intensive Care Med..

[95] Genc A., Ryk M., Suwala M. (2016). Ultrasound imaging in the general practitioner's office–a literature review. J. Ultrason..

[96] Mojoli F., Bouhemad B., Mongodi S. (2019). Lung ultrasound for critically ill patients. Am. J. Respir. Crit. Care Med..

[97] Buonsenso D., Pata D., Chiaretti A. (2020). COVID-19 outbreak: less stethoscope, more ultrasound. Lancet Respir. Med..

[98] Soldati G., Smargiassi A., Inchingolo R. (2020). Is there a role for lung ultrasound during the COVID-19 pandemic?. J. Ultrasound Med..

[99] Wang T., Zhang D., Wang L. (2018). Contactless respiration monitoring using ultrasound signal with off-the shelf audio devices. IEEE Internet Things J..

[100] Hilmizen N., Bustamam A., Sarwinda D. (2020). 2020 3rd International Seminar on Research of Information Technology and Intelligent Systems (ISRITI), December 10−11, 2020.

[101] Chandra T.-B., Verma K., Singh B.-K. (2021). Coronavirus disease (COVID-19) detection in chest X-ray images using majority voting-based classifier ensemble. Expert Syst. Appl..

[102] Ismael A.-M., Şengür A. (2021). Deep learning approaches for COVID-19 detection based on chest X-ray images. Expert Syst. Appl..

[103] Nayak S.-R., Nayak D.-R., Sinha U. (2021). Application of deep learning techniques for detection of COVID-19 cases using chest X-ray images: a comprehensive study. Biomed. Signal Process Control.

[104] Serte S., Demirel H. (2021). Deep learning for diagnosis of COVID-19 using 3D CT scans. Comput. Biol. Med..

[105] Shi F., Wang J., Shi J. (2020). Review of artificial intelligence techniques in imaging data acquisition, segmentation and diagnosis for COVID-19. IEEE Rev. Biomed. Eng..

[106] Ozsahin I., Sekeroglu B., Musa M.S. (2020). Review on diagnosis of COVID-19 from chest CT images using artificial intelligence. Comput. Math. Methods Med..

[107] Ahuja S., Panigrahi B.-K., Dey N. (2021). Deep transfer learning-based automated detection of COVID-19 from lung CT scan slices. Appl. Intell..

[108] Udugama B., Kadhiresan P., Kozlowski H.-N. (2020). Diagnosing COVID-19: the disease and tools for detection. ACS Nano.

[109] Shah S.-A., Yang X., Abbasi Q.-H. (2019). Cognitive health care system and its application in pill-rolling assessment. Int. J. Numer. Model. Electron. Network. Dev. Field..

[110] Yang X., Fan D., Ren A. (2020). Diagnosis of the hypopnea syndrome in the early stage. Neural Comput. Appl..

[111] Shah S.-A., Tahir A., Ahmad J. (2020). Sensor fusion for identification of freezing of gait episodes using Wi-Fi and radar imaging. IEEE Sensor. J..

[112] Ding C., Zou Y., Sun L. (2019). 2019 IEEE MTT-S International Wireless Symposium (IWS), May 19−22, 2019.

[113] Shah S.-A., Fioranelli F. (2019). 2019 International Radar Conference (RADAR), September 23−27, 2019.

[114] Fioranelli F., Le Kernec J., Shah S.-A. (2019). Radar for health care: recognizing human activities and monitoring vital signs. IEEE Potentials.

[115] Alizadeh M., Shaker G., De Almeida J.C.M. (2019). Remote monitoring of human vital signs using mm-wave FMCW radar. IEEE Acc..

[116] Gennarelli G., Ludeno G., Soldovieri F. (2016). Real-time through-wall situation awareness using a microwave Doppler radar sensor. Rem. Sens..

[117] Yang X., Shah S.-A., Ren A. (2018). Detection of essential tremor at the S-band. IEEE J. Transl. Eng. Heal. Med..

[118] Kushwaha S., Bahl S., Bagha A.K. (2020). Significant applications of machine learning for COVID-19 pandemic. J. Ind. Intg. Mgmt..

[119] Cai Q., Wang H., Li Z. (2019). A survey on multimodal data-driven smart healthcare systems: approaches and applications. IEEE Acc..

[120] Dinh A., Miertschin S., Young A. (2019). A data-driven approach to predicting diabetes and cardiovascular disease with machine learning. BMC Med. Inf. Decis. Making.

[121] Chang Y.-J., Hung K.-C., Wang L.-K. (2021). A real-time artificial intelligence-assisted system to predict weaning from ventilator immediately after lung resection surgery. Int. J. Environ. Res. Publ. Health.

[122] T. Ba, S. Li, Y. Wei, A data-driven machine learning integrated wearable medical sensor framework for elderly care service, Measurement 167 (2021), 108383.

[123] Bhavsar H., Ganatra A. (2012). A comparative study of training algorithms for supervised machine learning. Int. J. Soft Comput. Eng..

[124] Singh A., Thakur N., Sharma A. (2016). 2016 3rd Int. Conf. Comput. Sustain. Glob. Dev. (INDIACom), IEEE.

[125] Osisanwo F.Y., Akinsola J.E.T., Awodele O. (2017). Supervised machine learning algorithms: classification and comparison. Int. J. Comput. Trends Technol..

[126] Li X., Zhang D., Xiong J. (2018). Training-free human vitality monitoring using commodity Wi-Fi devices. Proc. ACM Interact. Mob. Wearable Ubiquitous Technol..

[127] Qian K., Wu C., Yang Z. (2018). Enabling contactless detection of moving humans with dynamic speeds using CSI. ACM Trans. Embed. Comput. Syst..

[128] Damodaran N., Haruni E., Kokhkharova M. (2020). Device free human activity and fall recognition using WiFi channel state information (CSI). CCF Trans. Pervasive Comput. Interact..

[129] Wang Z., Jiang K., Hou Y. (2019). A survey on CSI-based human behavior recognition in through-the-wall scenario. IEEE Acc..

[130] Guo L., Wang L., Lin C. (2019). Wiar: a public dataset for WiFi-based activity recognition. IEEE Acc..

[131] Aziz Shah S., Ahmad J., Tahir A. (2020). Privacy-preserving non-wearable occupancy monitoring system exploiting Wi-Fi imaging for next-generation body centric communication. Micromachines.

[132] Kim S.-C., Kim T.-G., Kim S.-H. (2019). 2019 International Conference on Artificial Intelligence in Information and Communication.

[133] Gu Y., Wang Y., Liu Z. (2020). SleepGuardian: an RF-based healthcare system guarding your sleep from Afar. IEEE Netw.

[134] Lee S., Park Y.-D., Suh Y.-J. (2018). 2018 15th IEEE Annual Consumer Communications & Networking Conference (CCNC), January 12−15, 2018, Las Vegas, NV, USA.

[135] Wang X., Yang C., Mao S. (2020). Resilient respiration rate monitoring with realtime bimodal CSI data. IEEE Sensor. J..

[136] Zhu S., Xu J., Guo H. (2018). 2018 IEEE International Conference on Communications (ICC), May 20−24, 2018.

[137] Çağlıyan B., Gürbüz S.Z. (2015). Micro-Doppler-based human activity classification using the mote-scale BumbleBee radar. IEEE. Geosci. Remote. Sens. Lett..

[138] Li W., Tan B., Piechocki R. (2018). Passive radar for opportunistic monitoring in E-health applications. IEEE J. Transl. Eng. Heal. Med..

[139] Taylor W., Shah S.-A., Dashtipour K. (2020). An intelligent non-invasive real-time human activity recognition system for next-generation healthcare. Sensors.

[140] Sigg S., Scholz M., Shi S. (2014). RF-sensing of activities from non-cooperative subjects in device-free recognition systems using ambient and local signals. IEEE Trans. Mobile Comput..

[141] Sharma S., Mohammadmoradi H., Heydariaan M. (2019).

[142] Kumar R., Arora R., Bansal V. (2020).

[143] Kassani S.-H., Kassasni P.-H., Wesolowski M.J. (2021). Automatic detection of coronavirus disease (COVID-19) in X-ray and CT images: a machine learning-based approach. Biocybern. Biomed. Eng..

[144] Saygili A. (2021). A new approach for computer-aided detection of coronavirus (COVID-19) from CT and X-ray images using machine learning methods. Appl. Soft Comput..

[145] Wu J., Zhang P., Zhang L. (2020).

[146] Brinati D., Campagner A., Ferrari D. (2020). Detection of COVID-19 infection from routine blood exams with machine learning: a feasibility study. J. Med. Syst..

[147] Khanday A.M.U.D., Rabani S.-T., Khan Q.-R. (2020). Machine learning based approaches for detecting COVID-19 using clinical text data. Int. J. Inf. Technol..

[148] Pahar M., Klopper M., Warren R. (2021). COVID-19 cough classification using machine learning and global smartphone recordings. Comput. Biol. Med..

[149] Khalifa N.E.M., Taha M.H.N., Hassanien A.-E. (2020). Detection of coronavirus (COVID-19) associated pneumonia based on generative adversarial networks and a fine-tuned deep transfer learning model using chest X-ray dataset. arXiv..

[150] Motamed S., Rogalla P., Khalvati F. (2021). RANDGAN: randomized generative adversarial network for detection of COVID-19 in chest X-ray. Sci. Rep..

[151] van der Schaar M., Alaa A.-M., Floto A. (2021). How artificial intelligence and machine learning can help healthcare systems respond to COVID-19. Mach. Learn..

[152] Goodfellow I.-J., Pouget-Abadie J., Mirza M. (2020). Generative adversarial networks, Commun. ACM.

[153] Choi E., Biswal S., Malin B. (2017). Proceedings of Machine Learning for Health Care 2017, Proceedings of Machine Research,.

[154] Iwendi C., Bashir A.-K., Peshkar A. (2020). COVID-19 patient health prediction using boosted random forest algorithm. Front. Public Health.

[155] Kaur P., Kumar R., Kumar M. (2019). A healthcare monitoring system using random forest and Internet of Things (IoT). Multimed. Tool. Appl..

[156] Simsekler M.C.E., Qazi A., Alalami M.-A. (2020). Evaluation of patient safety culture using a random forest algorithm. Reliab. Eng. Syst. Saf..

[157] Ogbuabor G., La R. (2018). Proceedings of the 2018 10th International Conference on Machine Learning and Computing.

[158] Amin M.-Z., Ali A. (2017).

[159] Krishna C.-L., Reddy P.V.S. (2019). 2019 3rd International Conference on Computing and Communications Technologies (ICCCT).

[160] Jan S.-U., Lee Y.-D., Shin J. (2017). Sensor fault classification based on support vector machine and statistical time-domain features. IEEE Acc..

[161] Ahmad Z., Rai A., Maliuk A.S. (2020). Discriminant feature extraction for centrifugal pump fault diagnosis. IEEE Acc..

[162] Jan S.-U., Lee Y.-D., Koo I.-S. (2021). A distributed sensor-fault detection and diagnosis framework using machine learning. Inf. Sci..

[163] Venkatesan C., Karthigaikumar P., Paul A. (2018). ECG signal preprocessing and SVM classifier-based abnormality detection in remote healthcare applications. IEEE Acc..

[164] Saeed U., Jan S.-U., Lee Y.-D. (2021). Fault diagnosis based on extremely randomized trees in wireless sensor networks. Reliab. Eng. Syst. Saf..

[165] Soltaninejad M., Yang G., Lambrou T. (2017). Automated brain tumour detection and segmentation using superpixel-based extremely randomized trees in FLAIR MRI. Int. J. Comput. Assist. Radiol. Surg..

[166] Padmaja B., Prasa V., Sunitha K. (2020). A novel random split point procedure using extremely randomized (extra) trees ensemble method for human activity recognition. EAI Endorsed Trans. Pervasive Heal. Technol..

[167] Saeed U., Lee Y.-D., Jan S.U. (2021). CAFD: context-aware fault diagnostic scheme towards sensor faults utilizing machine learning. Sensors (Basel).

[168] Xing W., Bei Y. (2019). Medical health big data classification based on KNN classification algorithm. IEEE Acc..

[169] Venkataramanaiah B., Kamala J. (2020). ECG signal processing and KNN classifier based abnormality detection by VH-doctor for remote cardiac healthcare monitoring. Soft Comput..

[170] Khateeb N., Usman M. (2017). BDIOT2017: International Conference on Big Data and Internet of Thing, London, UK.

